# Grip strength as a systems biomarker of aging: neuromuscular junction constraints and biomarker decoupling in gene therapy

**DOI:** 10.3389/fragi.2026.1908078

**Published:** 2026-08-27

**Authors:** Patrick E. Sewell, Christopher Jensen

**Affiliations:** Triple Helix Science | Triple Helix Oncology, Santa Ana, CA, United States

**Keywords:** biological aging, biomarker, dynapenia, follistatin, gene therapy, grip strength, neuromuscular junction, sarcopenia

## Abstract

Grip strength has emerged as one of the most robust predictors of mortality, disability and healthspan across populations. Yet its predictive validity rests on an often-unstated assumption: that grip strength serves as a passive readout of systemic biological integrity rather than an isolated measure of forearm function. Here we propose that grip strength derives its prognostic power from its position as a convergent output of multiple aging-sensitive physiological systems—including neuromuscular, vascular, metabolic, endocrine and inflammatory networks. We highlight the neuromuscular junction (NMJ) as a particularly critical and often-overlooked rate-limiting factor, noting that age-related strength loss (∼2.5–4% annually) outpaces mass loss (∼0.6–1% annually) by two-to fivefold—a disparity attributable in large part to NMJ deterioration. We introduce the concept of biomarker decoupling and apply it to the emerging landscape of longevity gene therapies being explored in early translational and compassionate-use settings—including follistatin, klotho, FOXO3, hTERT, SIRT1, PGC-1α, VEGF and FGF21. Critically, we argue that follistatin’s anabolic efficacy is contingent on intact NMJ integrity, with denervated muscle fibers exhibiting a blunted net anabolic response despite elevated follistatin expression—creating a therapeutic paradox wherein mass gains can occur without proportional functional improvement. We provide a conceptual analysis of how each therapy may influence grip strength, predict decoupling risk based on the breadth of systems affected, outline plausible timing windows for intervention, and propose a heuristic framework for clinical interpretation. The decoupling categories and any numeric ranges presented here are conceptual and hypothesis-generating rather than empirically validated.

## Introduction

Grip strength occupies a privileged position among biomarkers of biological aging. Meta-analyses encompassing hundreds of thousands of participants consistently demonstrate that grip strength predicts all-cause mortality with effect sizes comparable to established risk factors such as systolic blood pressure (Bohannon, 2019; Rantanen et al., 1999; Sayer and Kirkwood, 2015). This predictive relationship persists across age groups, ethnicities and disease states, extending beyond mortality to encompass cognitive decline, disability onset, hospitalization risk and quality of life measures (Rantanen et al., 1999; Visser et al., 2005). The measurement is inexpensive, non-invasive, and requires minimal training—qualities that have secured its place in geriatric assessment, sarcopenia diagnosis, and preoperative risk stratification worldwide (Manini and Clark, 2012; Schaap et al., 2006).

Yet the mechanistic basis for this remarkable predictive breadth remains incompletely understood. The dominant interpretation treats grip strength as a convenient proxy for overall muscle mass or general frailty. We argue this view is incomplete and increasingly problematic. When grip strength remains predictive after statistical adjustment for lean mass—while lean mass alone loses significance—something beyond simple muscularity must be at work.

This interpretive challenge becomes urgent as longevity medicine enters a new era. Longevity gene therapies are being explored in early translational and compassionate-use settings and include follistatin for muscle enhancement, klotho for multi-system protection, FOXO3 for stress resistance, hTERT for telomere extension, SIRT1 for metabolic regulation, PGC-1α for mitochondrial biogenesis, VEGF for vascular function, and FGF21 for metabolic health. These therapies represent a fundamental shift from observational aging assessment to interventional aging modification.

The central question this Hypothesis and Theory article addresses is: How should clinicians interpret grip strength changes in patients receiving longevity gene therapies? We propose that grip strength predicts mortality because it integrates information from multiple aging systems—and that understanding this integration is essential for interpreting grip changes in patients receiving targeted interventions.

Accordingly, this article is presented as a mechanistic and interpretive Hypothesis and Theory article, intended to clarify biomarker meaning and limitations rather than to provide clinical guidance or therapeutic endorsement.

## Epidemiological foundation and the mechanistic gap

The epidemiological evidence supporting grip strength as a mortality predictor is robust. The Prospective Urban Rural Epidemiology (PURE) study, following nearly 140,000 adults across 17 countries, found that each 5 kg reduction in grip strength increased cardiovascular mortality risk by 17% and all-cause mortality by 16% (Leong et al., 2015). UK Biobank data from half a million participants confirmed these associations across multiple disease endpoints (Celis-Morales et al., 2018). Crucially, these relationships persist after adjustment for body mass index, physical activity, and even lean muscle mass itself (Leong et al., 2015; Celis-Morales et al., 2018; Newman et al., 2006).

This last finding is telling. If grip strength were merely a proxy for muscle quantity, adjustment for lean mass should attenuate the association. That it does not suggests grip strength captures something about functional reserve that static measures miss. We propose this ‘something’ is the integrated function of multiple aging-sensitive systems, each required for maximal grip force generation.

Yet correlation does not imply mechanistic understanding. Without a causal framework, grip strength reveals risk but does not explain it—limiting its interpretive value precisely when patients receive interventions targeting specific aging pathways. A patient receiving follistatin gene therapy who shows grip improvement poses a critical question: does this improvement reflect reduced mortality risk, or has the intervention merely enhanced one component of a multi-system biomarker?

## The neuromuscular junction as rate-limiting factor

A striking observation in the aging literature has received insufficient attention in biomarker interpretation: age-related strength loss consistently outpaces mass loss by a factor of two to five (Clark and Manini, 2008; Goodpaster et al., 2006). A quantitative review of the aging skeletal-muscle literature reports that while muscle mass declines at approximately 0.6%–1% annually after age 50, grip strength declines at approximately 2.5%–4% annually (Mitchell et al., 2012); these are review-derived estimates rather than pooled meta-analytic effect sizes. Normative life-course data further show that grip strength peaks in early adulthood, is broadly maintained through midlife, and enters measurable decline from the mid-40s onward (Dodds et al., 2014). This disparity—termed dynapenia, the age-related loss of muscle strength that is disproportionate to any accompanying loss of muscle mass—indicates that neural and neuromuscular factors, rather than myofiber quantity alone, are primary determinants of functional strength loss.

The neuromuscular junction (NMJ) emerges as a critical site of age-related deterioration. By age 70, approximately 25% of NMJs demonstrate morphological evidence of partial denervation (Hepple and Rice, 2016). Estimates vary by muscle group, methodology, and species; however, convergent human and animal data consistently indicate substantial age-associated NMJ fragmentation and incomplete reinnervation by the seventh decade. These changes include fragmentation of the postsynaptic apparatus, reduced acetylcholine receptor density, withdrawal of terminal axon branches, and incomplete reinnervation following denervation events. Even partial NMJ dysfunction produces force deficits disproportionate to the extent of structural change, as a single motor neuron may control hundreds of muscle fibers.

This NMJ-centric view of strength decline has profound implications for gene therapy interpretation. Muscle mass interventions that do not address NMJ integrity face a fundamental ceiling on their capacity to restore function. A hypertrophied muscle fiber that lacks adequate innervation cannot contribute maximally to force production. The clinical correlate is a patient who gains mass but not proportional strength—a pattern we term the therapeutic paradox.

We propose that NMJ integrity should be conceptualized as a gating factor for anabolic therapies. Just as a narrow doorway limits the utility of a larger room, impaired NMJ function limits the functional benefit of enhanced muscle mass. This gating effect is not absolute—partial improvements remain possible—but it establishes an upper bound on grip strength gains achievable through muscle-targeted interventions alone.

## The systems integration model

**FIGURE 1:**
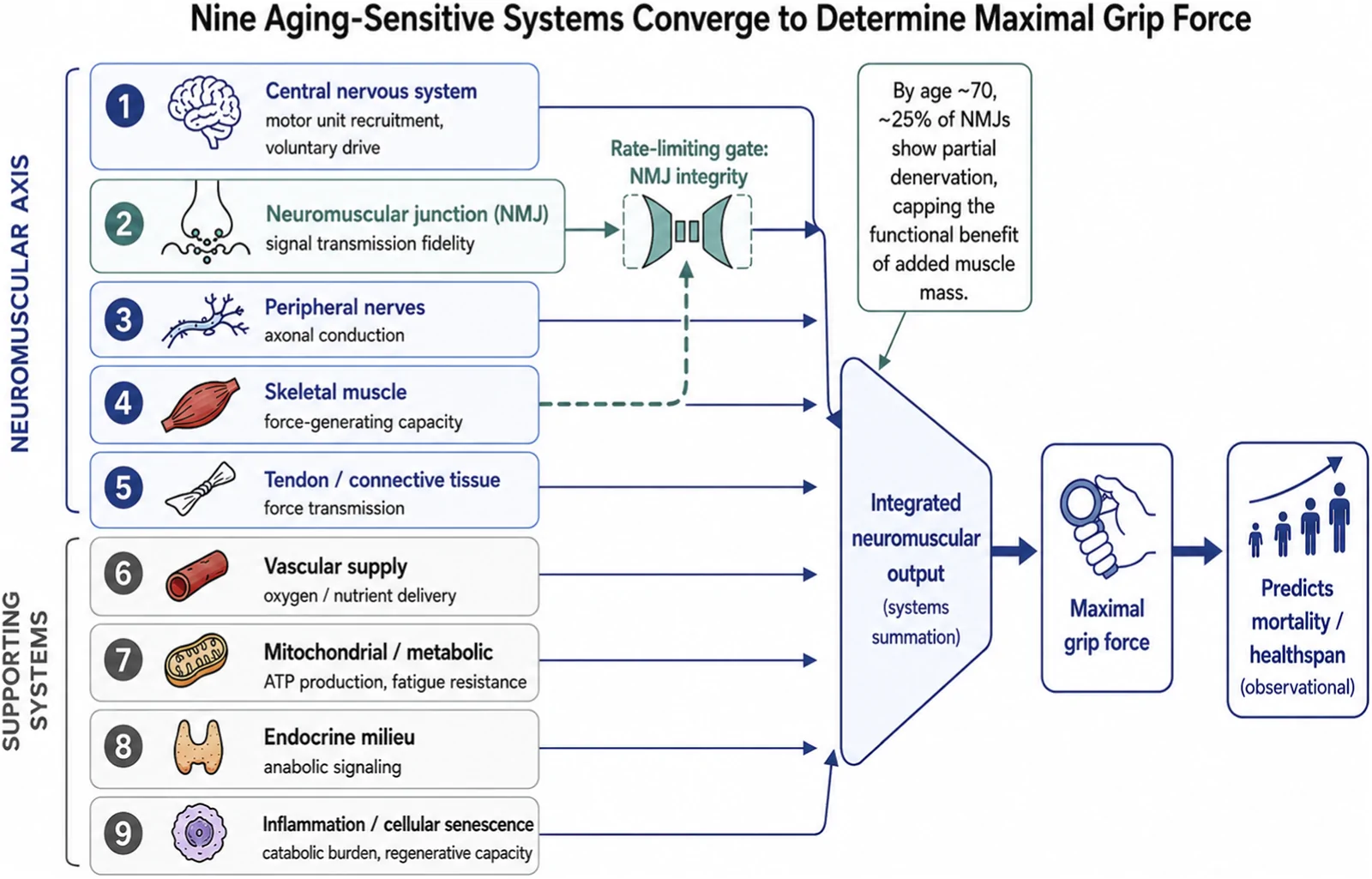
Grip strength as a convergent, multi-system output. Nine aging-sensitive physiological systems converge to determine maximal grip force. The systems are organized into a neuromuscular axis—central nervous system (motor unit recruitment and voluntary drive), neuromuscular junction (NMJ; signal transmission fidelity), peripheral nerves (axonal conduction), skeletal muscle (force-generating capacity), and tendon/connective tissue (force transmission)—and a set of supporting systems—vascular supply (oxygen and nutrient delivery), mitochondrial/metabolic function (ATP production and fatigue resistance), the endocrine milieu (anabolic signaling), and inflammation/cellular senescence (catabolic burden and regenerative capacity). Their combined contribution forms an integrated neuromuscular output (systems summation) that yields maximal grip force, which in turn predicts mortality and healthspan in observational cohorts. The NMJ functions as a rate-limiting gate through which skeletal-muscle force must pass (dashed path): because partial denervation affects an estimated ∼25% of NMJs by age 70, the functional benefit obtainable from added muscle mass is capped. Abbreviations: NMJ, neuromuscular junction.

**FIGURE 2:**
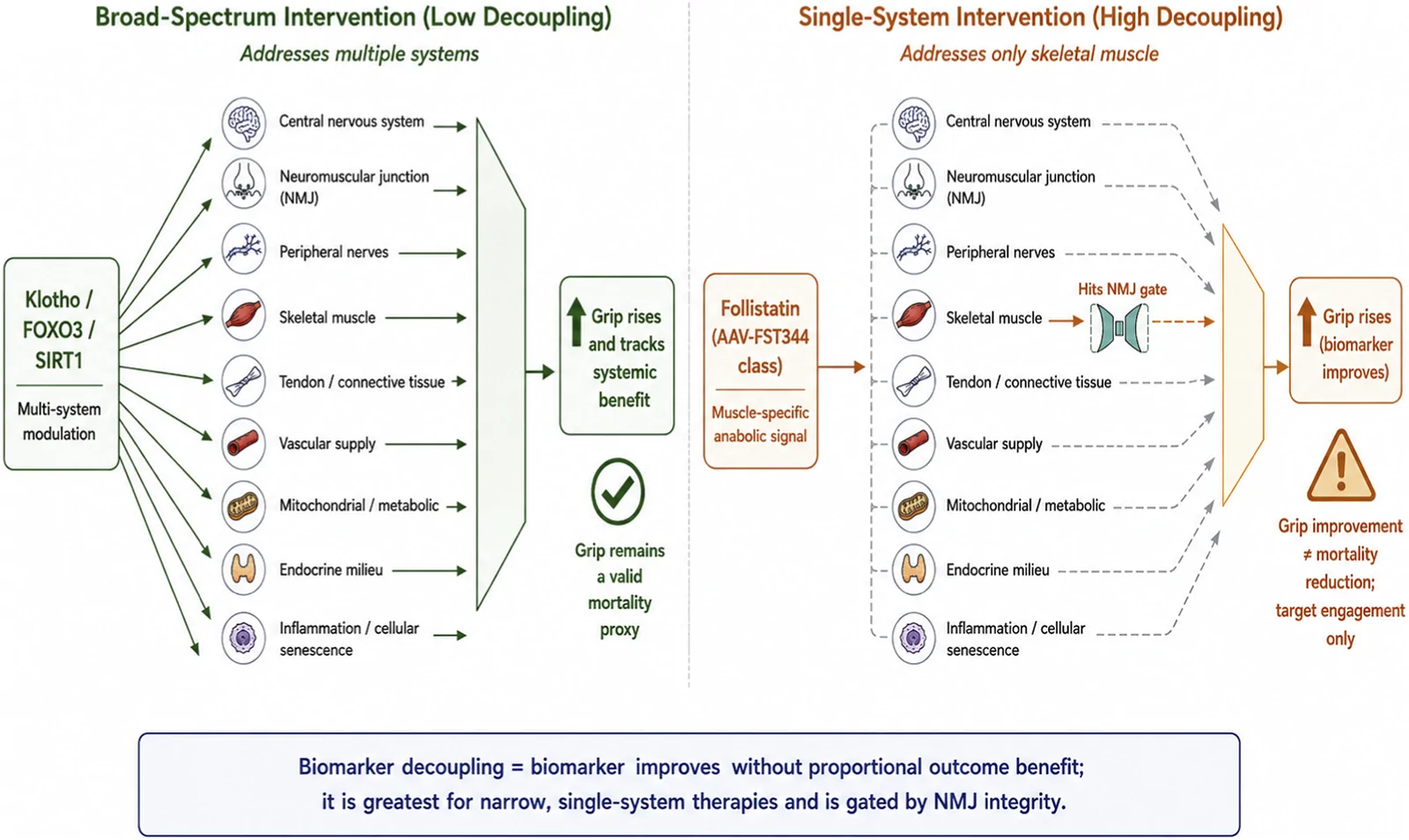
The origin of biomarker decoupling. The systems model explains why the prognostic validity of grip strength depends on the intervention. Left (low decoupling): a broad-spectrum intervention that modulates multiple systems simultaneously—illustrated by klotho, FOXO3, or SIRT1—raises grip strength in a manner that tracks systemic benefit, so grip remains a valid mortality proxy. Right (high decoupling): a single-system intervention acting only on skeletal muscle—illustrated by follistatin (AAV-FST344 class), a muscle-specific anabolic signal—raises grip strength while the remaining eight systems are left unaddressed (grey dashed paths), and its muscle-derived gain is further constrained at the NMJ gate; grip improves as a biomarker, but the change reflects target engagement rather than a proportional reduction in mortality risk. Biomarker decoupling—improvement of a biomarker without proportional outcome benefit—is therefore greatest for narrow, single-system therapies and is modulated by NMJ integrity. Abbreviations: NMJ, neuromuscular junction; AAV, adeno-associated virus; FST, follistatin.

Grip strength should be conceptualized as a compressed phenotype—a functional output arising from the coordinated performance of multiple physiological systems. Maximal grip force generation requires the simultaneous integrity of at least nine distinct biological layers, each deteriorating with age through partially independent mechanisms (Table 1). Figure 1 depicts these nine systems converging on grip force, with the neuromuscular junction positioned as a rate-limiting gate through which muscle-targeted signals must pass; Figure 2 contrasts a broad-spectrum intervention with a single-system intervention to show the point at which biomarker decoupling arises.

**TABLE 1:** Physiological systems contributing to grip strength and their relevant gene therapy targets.

System	Age-related changes	Grip contribution	Relevant gene therapies
Central nervous system	Motor cortex atrophy, reduced motor unit recruitment	Voluntary activation, coordination	Klotho (Kuro-o et al., 1997), FOXO3 (Willcox et al., 2008), SIRT1 (Willcox et al., 2008)
Neuromuscular junction*	Denervation, AChR loss, fragmentation	Signal transmission fidelity	Klotho (Kuro-o et al., 1997; Dubal et al., 2014), FOXO3 (Willcox et al., 2008)* (indirect)*
Peripheral nerves	Axonal degeneration, demyelination	Conduction velocity	Klotho (Kuro-o et al., 1997), hTERT (Bernardes de Jesus et al., 2012)
Skeletal muscle†	Fibre atrophy, type II loss, fat infiltration	Force generation capacity	Follistatin (McPherron et al., 1997; Latres et al., 2015), PGC-1α (Handschin and Spiegelman, 2008)
Vascular‡	Capillary rarefaction, endothelial dysfunction	Oxygen/nutrient delivery	VEGF (Celletti et al., 2001, Carmeliet, 2000), Klotho (Kuro-o et al., 1997), FGF21 (Geng et al., 2020)
Mitochondrial	Reduced biogenesis, oxidative damage	ATP production, fatigue resistance	PGC-1α (Handschin and Spiegelman, 2008, Lin et al., 2002), SIRT1 (Imai and Guarente, 2014)
Endocrine	Reduced anabolic hormones, insulin resistance	Anabolic signaling, metabolic support	FGF21 (Geng et al., 2020, Kharitonenkov and DiMarchi, 2015), SIRT1 (Imai and Guarente, 2014)
Inflammatory	Chronic low-grade inflammation	Catabolic burden, recovery capacity	FOXO3 (Willcox et al., 2008, Martins et al., 2016), Klotho (Kuro-o et al., 1997; Kuro-o, 2019), SIRT1 (Imai and Guarente, 2014)
Cellular senescence	Accumulation of senescent cells, SASP	Regenerative capacity, tissue quality	hTERT (Bernardes de Jesus et al., 2012, Mojiri et al., 2021, Bodnar et al., 1998, Morales et al., 1999, Jiang et al., 1999)

*Superscript numbers indicate reference citations.* NMJ, integrity is recognized as a rate-limiting factor; by age 70, ∼25% of NMJs, show partial denervation (Hepple and Rice, 2016).

^†^ Follistatin efficacy is contingent on adequate NMJ, integrity (Winbanks et al., 2015); see text.

^‡^ VEGF, effects are context-dependent; appropriate for ischemic disease, unfavorable risk-benefit for healthy longevity applications (Celletti et al., 2001; Carmeliet, 2000).*.

Central nervous system integrity determines motor unit recruitment patterns and the capacity for maximal voluntary effort. Neuromuscular junction function—now recognized as a distinct and critical layer—governs the fidelity of neural signal transmission to muscle fibers, with age-related NMJ deterioration explaining much of the disparity between mass and strength decline. Peripheral nerve function determines axonal health and conduction velocity. Skeletal muscle biology—including fiber type composition, cross-sectional area, and contractile protein quality—determines the force-generating capacity of activated motor units. Tendon and connective tissue mechanics influence the efficiency of force transmission from muscle to bone.

Beyond the neuromuscular axis, grip strength depends on adequate vascular supply to meet the metabolic demands of sustained contraction. Mitochondrial function and metabolic flexibility influence the energy available for both maximal efforts and recovery between contractions. The endocrine milieu—encompassing anabolic hormones, insulin sensitivity, and stress hormone regulation—shapes the chronic adaptations that maintain muscle mass and quality. Systemic inflammation, when chronically elevated, drives catabolic processes that erode grip strength independent of other factors. Finally, cellular senescence and telomere integrity affect the regenerative capacity of muscle and its supporting tissues.

## Biomarker decoupling: a framework for the gene therapy era

We introduce the term biomarker decoupling to describe the phenomenon whereby an intervention improves a biomarker that is observationally associated with an outcome, without proportionally improving the outcome itself. In the context of longevity gene therapy, decoupling occurs when a therapy improves grip strength through its target system while leaving other aging pathways—and therefore mortality risk—substantially unaddressed.

Decoupling is not inherently problematic—it simply means the biomarker’s interpretation must change. A patient receiving follistatin gene therapy who shows 20% grip improvement should not be told their mortality risk has decreased by the amount observational data would predict for a 20% natural grip increase. The improvement reflects target engagement, not necessarily systemic benefit.

### Quantifying decoupling: a conceptual framework

We propose a conceptual decoupling ratio for quantifying this phenomenon:

*Decoupling Ratio* = (*Observed Δ Grip/Expected Δ Grip*) ÷ (*Observed Δ Mortality/Expected Δ Mortality*).

This formulation is intended as a heuristic framework rather than a validated quantitative model, providing conceptual guidance for interpreting biomarker–outcome divergence rather than a calculable clinical metric. All numerical ranges presented here, including the proposed thresholds and the therapy-specific decoupling estimates, are mechanistically informed estimates and should be treated as hypotheses to be tested, not as empirically derived effect sizes. Accordingly, we recommend interpreting decoupling primarily on an ordinal scale—low, moderate, or high—reflecting how many of the grip-contributing systems an intervention engages; the numeric ranges given below are illustrative anchors rather than calculable values.

The mathematical logic underlying this ratio derives from the fundamental assumption of observational epidemiology: that grip strength predicts mortality because it reflects the integrated function of multiple aging-sensitive systems. When grip strength declines naturally—through sedentary behavior, chronic disease, or biological aging—this decline typically reflects concurrent deterioration across neural, muscular, vascular, metabolic, and inflammatory systems. The mortality prediction emerges from this multi-system decline, not from grip strength *per se*.

The expected relationships can be derived from observational data. The PURE study established that each 5 kg reduction in grip strength associates with a 16% increase in all-cause mortality risk (Leong et al., 2015). UK Biobank data confirm similar effect sizes across diverse populations (Celis-Morales et al., 2018). These observational relationships provide the denominator expectations: if a 10% natural grip improvement associates with (for example,) an 8% mortality reduction in observational cohorts, this establishes the ‘expected’ coupling ratio.

When a therapeutic intervention improves grip strength through a targeted mechanism, the numerator captures the actual observed changes. If follistatin therapy produces a 20% grip improvement but addresses only muscular hypertrophy without affecting neural integrity, vascular function, or inflammatory burden, the observed mortality benefit will be substantially less than observational data would predict for a 20% natural grip increase.

A ratio of 1.0 indicates preserved coupling—the intervention has produced grip improvement through mechanisms that fully recapitulate the multi-system integration that underlies observational predictions. This would be expected for interventions affecting all nine systems contributing to grip strength, or for broad lifestyle interventions like comprehensive exercise programs that simultaneously improve neural function, muscle quality, vascular health, and metabolic flexibility.

A ratio >1.5 indicates meaningful decoupling—grip improvement exceeds mortality benefit by a margin that has clinical significance. The 1.5 threshold is proposed based on clinical utility: smaller discrepancies (1.1–1.4) may fall within measurement error and natural variation, while ratios approaching 2.0 or higher suggest the intervention has produced substantial biomarker improvement without proportional systemic benefit. This is conceptually analogous to—though not equivalent to—the correlation-based criteria used in surrogate-endpoint validation, where regulatory agencies typically require strong surrogate–outcome correlation (*r* ^2^ > 0.5) (Prentice, 1989; Fleming and Powers, 2012); a decoupling ratio and a correlation coefficient are distinct metrics, and the parallel is offered as an analogy rather than an equivalence.

Different gene therapies have different predicted decoupling ratios based on how many systems they affect. Follistatin, targeted to act almost exclusively on skeletal muscle, would be expected to show high decoupling ratios (estimated 2.0–3.0) because grip improvement occurs through a single system while mortality risk depends on nine. Klotho, affecting neural, vascular, inflammatory, and endocrine systems simultaneously, would be expected to show lower decoupling ratios (estimated 1.0–1.3) because its multi-system effects more closely recapitulate the integrated decline that underlies observational predictions.

### Limitations and prospective validation requirements

This decoupling ratio is explicitly conceptual and requires prospective validation. Current longevity gene therapies lack the mortality outcome data needed to calculate actual ratios—validation will require registry-based longitudinal follow-up correlating biomarker changes with hard endpoints. The ratio also assumes linear relationships between grip changes and mortality changes, which may not hold across all effect sizes. Furthermore, interaction effects between systems may produce non-additive mortality relationships that complicate ratio interpretation.

Despite these limitations, the framework provides clinical utility by establishing a quantitative vocabulary for discussing the expected relationship between biomarker improvement and outcome benefit. Clinicians can use predicted decoupling ratios to set appropriate patient expectations and design complementary biomarker panels that triangulate multi-system assessment.

## NMJ integrity as a decoupling mechanism

The NMJ framework introduces a distinct mechanism for biomarker decoupling beyond the systems-breadth considerations already discussed. Even within the muscular system itself, interventions that increase mass without addressing innervation demonstrate intrasystem decoupling—grip improvement that fails to reflect the full functional benefit implied by mass gains.

Evidence for this phenomenon comes from studies of anabolic interventions in older adults. Testosterone supplementation, myostatin inhibition, and resistance training all demonstrate attenuated strength gains relative to mass gains in older compared to younger individuals (Rygiel et al., 2016). The common denominator is age-related NMJ deterioration: hypertrophied muscle fibers cannot contribute fully to force production if their innervation is compromised.

This creates a therapeutic paradox: interventions that successfully increase muscle mass may produce disappointing functional outcomes in individuals with advanced NMJ deterioration. Mass gains without functional improvement is not treatment failure per se—it represents target engagement limited by an unaddressed bottleneck. Recognizing this distinction is essential for appropriate patient selection and expectation management.

## The longevity gene therapy landscape

At least eight molecular targets in the Longevity gene therapy landscape are being explored in early translational and compassionate-use settings. Each targets distinct aspects of the aging process, and each has different implications for grip strength interpretation. We analyze each in turn, with particular attention to NMJ dependencies where relevant.

### Follistatin: myostatin inhibition and the NMJ constraint

Follistatin inhibits myostatin and activins, removing the endogenous brake on muscle growth. It produces robust increases in muscle mass and strength through enhanced myofiber hypertrophy and satellite cell activation (McPherron et al., 1997; Latres et al., 2015). The expected grip effect is an estimated 15%–30% improvement based on preclinical and early clinical data; this range is a literature-informed estimate rather than a measured effect size.

Follistatin gene therapy is an investigational therapy with peer-reviewed human trial data in muscle-wasting disease. Non-human-primate work demonstrated sustained expression and muscle-volume gains after intramuscular AAV delivery of the human follistatin-344 isoform (Kota et al., 2009), and early-phase trials in Becker muscular dystrophy and sporadic inclusion body myositis reported sustained transgene expression and functional signals in open-label cohorts (Mendell et al., 2015; Mendell et al., 2017). It is also under development for cancer-associated cachexia, a high-mortality syndrome that affects the majority of patients with advanced cancer and lacks any approved anabolic therapy (Baracos et al., 2018), which is the indication for which the authors’ oncology program (Triple Helix Oncology) developed it. One author’s company is developing an AAV-follistatin construct (AAV-FST344). We distinguish these disease indications—where the therapeutic endpoint is muscle mass and function directly—from the therapy’s off-label use for longevity or cosmetic muscle enhancement, where grip change may be misinterpreted as a mortality-risk surrogate; the biomarker-decoupling concern developed here applies principally to the latter context.

However, follistatin’s efficacy as a functional enhancer is critically dependent on neuromuscular junction integrity. Evidence from preclinical models indicates that denervated or partially denervated muscle fibers exhibit a markedly blunted net anabolic response to follistatin (Winbanks et al., 2015). The mechanism involves activity-dependent signaling: without adequate neural input, the mechanical loading signals that potentiate anabolic pathways are attenuated, and newly synthesized contractile proteins cannot be appropriately organized into functional sarcomeric units. In a controlled study of AAV-delivered follistatin, protein synthesis increased in both innervated and denervated muscle, but protein degradation was reduced only in innervated muscle, and follistatin delivered at the time of denervation did not prevent subsequent wasting (Winbanks et al., 2015); the precise magnitude of this attenuation in humans is not established. Reference to a downstream BMP-signaling mechanism is retained for context (Sartori et al., 2013).

This NMJ dependency creates a ceiling effect for follistatin therapy that varies with patient age and baseline neural status. In younger individuals with intact NMJs, follistatin can produce substantial and functional hypertrophy. In older individuals with significant NMJ deterioration, the same intervention may produce mass gains that translate poorly to functional improvement—the therapeutic paradox discussed above.

#### Timing window

Based on the kinetics of NMJ deterioration and follistatin’s mechanism, we hypothesize an intervention window of approximately ages 50–55, when sarcopenic processes are beginning but NMJ integrity remains largely preserved (<5% denervation) (Hepple and Rice, 2016). This window is motivated by the convergence of the onset-of-decline literature—measurable grip decline from the mid-40s (Dodds et al., 2014) and accelerating strength loss after age 50 (12)—with the preservation of NMJ integrity at 50–55 (14). Pre-emptive follistatin therapy during this window may achieve both larger magnitude effects and better functional translation than delayed intervention after NMJ deterioration has progressed. After age 65–70, when ∼25% of NMJs show partial denervation (Hepple and Rice, 2016), follistatin’s efficacy as a functional enhancer is substantially constrained, and mass gains may translate poorly to functional strength gains (Winbanks et al., 2015). These age bands are hypothesis-generating approximations rather than validated clinical thresholds. These age bands are approximations based on aggregate human and animal data; comorbidities, lifelong physical activity, and genetic factors may shift NMJ deterioration by several years in either direction.

Beyond NMJ considerations, follistatin acts almost exclusively on skeletal muscle, with limited direct effects on neural, vascular, metabolic, or inflammatory systems (McPherron et al., 1997; Latres et al., 2015). This narrow targeting creates high decoupling risk (predicted ratio 2.0–3.0). A patient receiving follistatin may demonstrate substantial grip gains while retaining the full complement of neural degeneration, vascular dysfunction, metabolic inflexibility, and inflammatory burden that collectively drive mortality risk.

#### Clinical interpretation

In follistatin-treated patients, grip strength loses validity as a mortality proxy. Grip improvement should be interpreted as target engagement, not mortality risk reduction. The magnitude of functional improvement should be evaluated against baseline NMJ status where assessable. Patients showing mass gains without proportional strength gains may have NMJ-limited response. Complementary biomarkers—gait speed, VO_2_max, inflammatory markers, cognitive testing—become essential for comprehensive risk assessment.

### Klotho: multi-system longevity factor with neuroprotective effects

Klotho is an anti-aging protein that functions as a circulating hormone and co-receptor for FGF23. Its effects are pleiotropic: suppression of oxidative stress and inflammation, enhancement of vascular function, protection against neurodegeneration, and modulation of mineral metabolism (Kuro-o et al., 1997; Kuro-o, 2019). Notably, klotho demonstrates neuroprotective effects that may extend to preservation of NMJ integrity—a property supported by its role in maintaining synaptic function and axonal health in both central and peripheral nervous systems (Dubal et al., 2014; Yokoyama et al., 2015). This distinguishes klotho from muscle-specific interventions.

The systems affected include neural function (neuroprotection, cognitive preservation, potentially NMJ maintenance), vascular health (endothelial function, arterial stiffness reduction), inflammatory tone (suppression of pro-inflammatory cytokines), and endocrine signaling (FGF23/phosphate axis modulation) (Kuro-o et al., 1997; Kuro-o, 2019). The expected grip effect is moderate (5%–15%) through multi-system enhancement rather than direct muscle effects (Yokoyama et al., 2015; Semba et al., 2011). This estimate derives from klotho’s indirect mechanisms: improved vascular delivery enhances muscle oxygenation during effort; reduced inflammation decreases catabolic burden; neuroprotection preserves motor unit recruitment capacity.

Importantly, klotho demonstrates low decoupling risk (predicted ratio 1.0–1.3). Because its effects span multiple systems that contribute to grip strength—including the neural components often missed by muscle-targeted therapies—improvement more accurately reflects genuine systemic benefit.

#### Clinical interpretation

Grip strength retains reasonable validity in klotho-treated patients. The magnitude of grip change may actually underestimate systemic benefit given the indirect nature of klotho’s muscle effects. Klotho may be particularly valuable as a combination therapy with muscle-targeted interventions, potentially preserving the NMJ integrity required for follistatin efficacy. Recommended complementary markers include cognitive function testing, vascular stiffness assessment (pulse wave velocity), and phosphate/calcium metabolism.

### FOXO3: stress resistance and longevity

FOXO3 is the transcription factor most consistently associated with extreme longevity in human genetic studies (Willcox et al., 2008; Martins et al., 2016). It regulates genes involved in oxidative stress resistance, autophagy, metabolism, and cell cycle arrest. FOXO3 activation promotes cellular maintenance and stress resilience across multiple tissues, including motor neurons (Willcox et al., 2008; Martins et al., 2016; Webb and Brunet, 2014).

The systems affected are broad: muscle (autophagy, protein quality control), neural (neuroprotection, motor neuron maintenance), vascular (endothelial stress resistance), metabolic (glucose homeostasis), inflammatory (cytokine regulation), and cellular senescence (cell cycle control) (Willcox et al., 2008; Martins et al., 2016). Expected grip improvement is moderate (5%–15%) with low decoupling risk (predicted ratio 1.0–1.4) (Webb and Brunet, 2014; Morris et al., 2015). The effect size estimate derives from FOXO3’s role in maintaining cellular quality across tissues: enhanced autophagy improves myofiber protein turnover; neuroprotection preserves motor neuron function; stress resistance reduces accumulated cellular damage.

FOXO3’s neuroprotective effects may help preserve NMJ function through maintenance of motor neuron health and axonal transport mechanisms (Webb and Brunet, 2014), making it a potentially synergistic combination partner for muscle-targeted therapies.

#### Clinical interpretation

Grip strength likely retains validity in FOXO3-treated patients. Improvement suggests genuine multi-system benefit consistent with the gene’s association with human longevity. Complementary markers include oxidative stress indicators (8-OHdG, F2-isoprostanes), autophagy markers where available, and glucose homeostasis measures (HbA1c, fasting glucose).

### hTERT and cancer risk: evidence and uncertainties

hTERT is the catalytic subunit of telomerase, the enzyme that extends telomeres. Telomere shortening limits cellular replicative capacity and is associated with aging and age-related diseases. Gene therapy aims to restore telomere length and extend cellular lifespan (Bernardes de Jesus et al., 2012; Mojiri et al., 2021).

The evidence bearing on hTERT and cancer risk must be carefully bounded. The landmark studies demonstrating that ectopic hTERT expression in normal human cells restores telomerase activity, extends telomeres, and prolongs cellular lifespan without inducing malignant transformation are *in vitro* immortalization studies of normal human cells (Bodnar et al., 1998; Morales et al., 1999; Jiang et al., 1999); the principal *in vivo* evidence of delayed aging without increased cancer derives from a mouse study (Bernardes de Jesus et al., 2012). None of these establishes the long-term oncologic safety of *in vivo* hTERT gene therapy in humans, and long-term human cancer-incidence data are not yet available; any statement of safety therefore applies under specific delivery conditions and remains to be confirmed in humans. Within those bounds, a distinction should be made: while elevated hTERT expression is detectable in the great majority of cancers, cancer cells manipulate endogenous hTERT expression through acquired promoter mutations (particularly at positions −124 and −146, creating novel ETS2 binding sites), and in these models hTERT expression does not cause normal cells to become cancerous (Bodnar et al., 1998; Morales et al., 1999; Jiang et al., 1999). Exogenous hTERT delivery via gene-therapy vectors is mechanistically different from endogenous promoter activation in cancer, and has not conferred oncogenic transformation in normal cells in the preclinical models examined.

Indeed, preclinical models suggest that hTERT-based interventions may, in principle, lower cancer-promoting signals through their effects on cellular senescence. Telomere shortening creates genomically unstable cells vulnerable to mutation; reversal of telomere shortening reduces this mutagenic substrate (Morales et al., 1999; Jiang et al., 1999). Multiple studies demonstrate that elevating TERT expression reduces senescence markers, inflammaging, and SASP (senescence-associated secretory phenotype)—the pro-inflammatory secretome that promotes cancer-permissive microenvironments (Morales et al., 1999; Jiang et al., 1999; Jaskelioff et al., 2011). However, long-term cancer incidence data in humans receiving hTERT gene therapy are not yet available.

The systems potentially affected are broad—all tissues with proliferative capacity could benefit through enhanced stem cell function and tissue regeneration, including satellite cells in muscle and Schwann cells supporting motor neurons. The expected grip effect is likely delayed, occurring through enhanced satellite cell function and tissue regeneration over months to years.

Decoupling risk is variable. If hTERT successfully extends telomeres across multiple tissues, grip improvement should reflect systemic benefit. If effects are tissue-specific or do not translate to functional improvement, some decoupling may occur.

#### Clinical interpretation

Long-term trajectory may be more informative than acute changes given the indirect mechanism of action. Monitor telomere length (leukocyte TL or tissue-specific where available) alongside grip to assess target engagement. The historical concern regarding oncogenic risk is not supported by current mechanistic and preclinical data under specific delivery conditions, though long-term human safety remains to be defined (Bernardes de Jesus et al., 2012; Bodnar et al., 1998; Morales et al., 1999; Jiang et al., 1999); long-term registry data will be valuable for confirming safety profiles in larger human cohorts.

### SIRT1: metabolic regulation and stress response

SIRT1 is an NAD^+^-dependent deacetylase that regulates metabolism, stress responses, and longevity pathways. It activates PGC-1α (mitochondrial biogenesis), FOXO transcription factors (stress resistance), and modulates inflammatory signaling. SIRT1 mediates many benefits of caloric restriction (Imai and Guarente, 2014; Satoh et al., 2013).

The systems affected are broad: metabolic (glucose homeostasis, lipid metabolism), mitochondrial (via PGC-1α activation), inflammatory (NF-κB suppression), neural (neuroprotection through deacetylation of key substrates), vascular (endothelial function, NO bioavailability), and muscle (oxidative capacity, fiber quality) (Imai and Guarente, 2014; Satoh et al., 2013; Cantó et al., 2009). Expected grip improvement is moderate (5%–15%) with low to moderate decoupling risk (predicted ratio 1.1–1.5) (Cantó et al., 2009; Gurd, 2011). The effect size derives from SIRT1’s metabolic effects: improved mitochondrial function enhances muscle energetics; reduced inflammation decreases catabolic signaling; enhanced insulin sensitivity improves anabolic responsiveness.

#### Clinical interpretation

Grip strength likely retains validity in SIRT1-treated patients. The degree of benefit may depend on baseline metabolic status and NAD^+^ availability. Pair with metabolic markers (HbA1c, lipid profile, NAD^+^ levels where measurable) to assess concordance.

### PGC-1α: mitochondrial biogenesis

PGC-1α is the master regulator of mitochondrial biogenesis. It increases mitochondrial content, enhances oxidative capacity, and improves metabolic flexibility. PGC-1α overexpression in muscle increases endurance capacity and resistance to fatigue (Handschin and Spiegelman, 2008; Lin et al., 2002).

The systems affected are primarily metabolic/mitochondrial, with secondary effects on muscle fiber type (promoting oxidative slow-twitch fibers) and potentially vascular function (through VEGF induction) (Handschin and Spiegelman, 2008; Lin et al., 2002). Neural, inflammatory, and endocrine systems are less directly affected—importantly, PGC-1α does not address NMJ deterioration. Expected grip improvement is moderate (5%–15%), primarily through enhanced fatigue resistance rather than peak force—sustained grip may improve more than maximal grip (Gill et al., 2018; Calvo et al., 2008). This prediction derives from PGC-1α′s mechanism: increased mitochondrial density improves ATP regeneration during repeated contractions; enhanced oxidative fiber proportion shifts the muscle toward fatigue-resistant phenotype.

Decoupling risk is moderate (predicted ratio 1.3–1.8). PGC-1α targets a specific system (mitochondrial) that has broad physiological importance, but neural degeneration, NMJ deterioration, inflammatory burden, and structural muscle changes remain unaddressed.

Clinical interpretation: Grip improvement suggests enhanced metabolic capacity but does not rule out ongoing deterioration in other systems. Pair with VO_2_max or exercise tolerance testing to assess concordance. Consider that sustained grip tasks may show greater improvement than single maximal efforts.

### VEGF: angiogenesis and context-dependent vascular effects

VEGF promotes angiogenesis and enhances vascular function. Gene therapy aims to increase capillary density, improve oxygen and nutrient delivery, and restore endothelial function. Naked plasmid VEGF165 gene transfer has been evaluated in controlled trials of peripheral arterial and critical limb ischemia encompassing several hundred patients (Celletti et al., 2001; Isner et al., 1996; Baumgartner et al., 1998), with a substantially larger post-marketing/registry experience—210 patients, predominantly in Russia and Eastern Europe—reflecting transient intramuscular delivery in documented ischemic populations rather than controlled-trial exposure (Deev et al., 2017). The registry figure is a post-marketing statistic, not a controlled-trial result (Ylä-Herttuala et al., 2017).

VEGF exemplifies a molecule with fundamentally dual biological effects that depend critically on tissue context (Celletti et al., 2001; Carmeliet, 2000). In ischemic tissues, VEGF restores perfusion through neovascularization and supports tissue survival—this is the therapeutic rationale for peripheral arterial disease applications. However, in non-ischemic or pathological tissues, uncontrolled VEGF expression raises concerns: destabilization of atherosclerotic plaques through vasa vasorum proliferation (Celletti et al., 2001), retinal neovascularization in predisposed individuals (diabetic retinopathy, wet AMD), and provision of angiogenic support to dormant tumors. These concerns apply particularly to unmodified (‘naked’) VEGF constructs that lack vessel stabilization co-factors (Angiopoietin-1/Tie2 axis), hypoxia-responsive expression gating, and tissue detargeting (Celletti et al., 2001; Carmeliet, 2000).

The systems affected are primarily vascular/microcirculatory, with secondary benefits to any tissue dependent on oxygen delivery (Ylä-Herttuala et al., 2017). Neural drive, NMJ function, muscle hypertrophy, metabolic regulation, and inflammatory tone are not directly affected. Expected grip improvement is modest (5%–10%), primarily through enhanced oxygen delivery during sustained effort (Chinsomboon et al., 2009).

Decoupling risk is moderate to high (predicted ratio 1.5–2.0). VEGF addresses vascular function but leaves most grip-contributing systems—including the critical NMJ—unaddressed. Risk-benefit considerations differ substantially between ischemic disease (where benefits may outweigh risks) and healthy longevity applications (where documented ischemia is absent and risk-benefit is unfavorable) (Celletti et al., 2001; Carmeliet, 2000).

#### Clinical interpretation

VEGF gene therapy is best suited for documented ischemic conditions where potential benefits outweigh known risks. For healthy longevity applications without documented ischemia, the risk-benefit profile is unfavorable (Celletti et al., 2001; Carmeliet, 2000). Improvement in ischemic patients suggests enhanced vascular delivery. Future VEGF constructs incorporating ischemia-responsive gating, vessel stabilization elements, and tissue detargeting may alter this calculus, but such systems require human validation.

### FGF21: metabolic hormone

FGF21 regulates glucose and lipid metabolism, enhances insulin sensitivity, and promotes energy expenditure. It is induced by fasting and may mediate some benefits of caloric restriction. FGF21 also has direct effects on vascular function and crosses the blood–brain barrier (Geng et al., 2020; Kharitonenkov and DiMarchi, 2015).

The systems affected include metabolic (glucose homeostasis, lipid oxidation, insulin sensitivity), vascular (endothelial function), inflammatory (through adipokine modulation and reduced ectopic lipid), and potentially neural (direct CNS effects via BBB penetration) (Geng et al., 2020; Kharitonenkov and DiMarchi, 2015; Markan et al., 2014). Expected grip improvement is modest (3%–10%) through improved metabolic function and vascular health, possibly improving muscle quality (reduced intramuscular fat infiltration) rather than mass (Markan et al., 2014; Tezze et al., 2019). This estimate derives from FGF21’s metabolic mechanism: improved insulin sensitivity enhances anabolic signaling; reduced intramuscular lipid improves contractile efficiency; better glucose utilization supports muscle energetics.

Decoupling risk is low to moderate (predicted ratio 1.1–1.5). FGF21’s multi-system effects and endogenous role in adaptive metabolism suggest grip changes may reasonably reflect systemic metabolic improvement.

#### Clinical interpretation

Grip strength likely retains validity in FGF21-treated patients, particularly when paired with metabolic markers. Consider grip/weight ratio given potential body composition changes. Recommended complementary markers include metabolic panel (glucose, HbA1c, lipids, liver enzymes), body composition assessment, and inflammatory markers (CRP, IL-6).

## Comprehensive decoupling predictions

Table 2 summarizes the predicted decoupling profiles for current longevity gene therapies based on the systems-integration model, with specific attention to NMJ dependency.

**TABLE 2:** Gene therapy targets, systems breadth, NMJ dependency, and decoupling risk assessment.

Therapy	Systems breadth	NMJ effect	Expected Δ Grip	Estimated decoupling range	Grip validity
Follistatin*	Narrow (muscle)	Dependent	15%–30%	2.0–3.0	Low (target engagement only)
Klotho	Broad	Potentially protective	5%–15%	1.0–1.3	High (multi-system)
FOXO3	Broad	Potentially protective	5%–15%	1.0–1.4	High (multi-system)
hTERT	Broad (proliferative)	Neutral to beneficial	Variable	1.2–1.6	Moderate (time-dependent)
SIRT1	Broad	Neutral	5%–15%	1.1–1.5	Moderate–High
PGC-1α	Moderate	Neutral	5%–15%	1.3–1.8	Moderate (metabolic bias)
VEGF†	Narrow (vascular)	Neutral	5%–10%	1.5–2.0	Low–Moderate
FGF21	Moderate	Neutral	3%–10%	1.1–1.5	Moderate–High

*Breadth indicates how many grip-contributing systems are affected. NMJ, effect indicates whether the therapy addresses, depends on, or is neutral to neuromuscular junction integrity. Predicted ratio = estimated decoupling ratio; values* ***>*** *1.5 indicate meaningful decoupling*. Follistatin efficacy is contingent on intact NMJ; patients with **>**10% denervation may show blunted functional response (Barns et al., 2014).

† VEGF, validity is context-dependent; appropriate for ischemic disease, unfavorable for healthy longevity applications (Celletti et al., 2001; Carmeliet, 2000). Grip validity indicates reliability of grip as a mortality proxy post-treatment. All ranges are conceptual estimates based on systems breadth and mechanism, not empirical measurements.*.

### Timing windows and intervention strategy

The NMJ constraint introduces timing considerations that extend beyond traditional views of ‘earlier is better.’ Because NMJ integrity declines progressively with age (Hepple and Rice, 2016), the therapeutic window for maximal benefit from muscle-targeted interventions is bounded.

#### Pre-emptive intervention (ages 50–55)

NMJ deterioration is minimal (<5% denervation). Follistatin and other anabolic therapies can achieve their full potential with good mass-to-function translation (Hepple and Rice, 2016; Barns et al., 2014). This window represents the optimal opportunity for pre-emptive muscle preservation before the compounding effects of NMJ loss, mitochondrial dysfunction, and inflammatory burden become established.

#### Intermediate window (ages 55–65)

NMJ deterioration is progressing (5%–15% denervation). Anabolic therapies retain efficacy but may show attenuated functional translation (Hepple and Rice, 2016). Combination approaches that include neuroprotective elements (klotho, FOXO3) may help preserve remaining NMJ function. Monitoring the mass-to-strength ratio provides insight into NMJ constraint.

#### Late intervention (ages 65+)

NMJ deterioration is substantial (∼25% denervation by age 70) (Hepple and Rice, 2016). The ceiling effect on anabolic therapy efficacy becomes pronounced. Mass gains may occur without proportional strength gains—the therapeutic paradox. At this stage, multi-system interventions (klotho, FOXO3, SIRT1) that address neural preservation, inflammation, and metabolism may provide better risk-to-benefit profiles than muscle-targeted approaches alone.

These timing considerations suggest that patient selection and intervention sequencing require individualized assessment. A 52-year-old with early sarcopenic changes and preserved neural function would, on this framework, be predicted to translate anabolic therapy into function more efficiently than an older individual with established denervation; this is a mechanistic hypothesis rather than a treatment recommendation. A 70-year-old with established NMJ deterioration may benefit more from multi-system approaches that address the neural and inflammatory factors limiting muscle function. These age bands are conceptual approximations rather than validated thresholds; individual NMJ trajectories vary with comorbidity, physical activity, and genetics, and prospective age-stratified data would be required before applying such windows in clinical trial design.

### Clinical interpretation framework

We propose the following framework for interpreting grip strength in gene therapy patients.

#### Document intervention history

Record all gene therapies received, dates, and doses. This information fundamentally changes grip interpretation.

#### Assess NMJ status where possible

Clinical indicators of NMJ function include the relationship between mass and strength (poor mass-to-strength ratio suggests NMJ limitation), response to prior anabolic interventions, and neurophysiological testing where available. Patients with suspected NMJ deterioration may have limited response to muscle-targeted therapies.

#### Assess systems breadth

Determine whether the patient’s gene therapy portfolio addresses narrow or broad aging systems using the framework above.

#### Consider timing relative to age

Earlier intervention (ages 50–55) with muscle-targeted therapies is likely to produce better functional outcomes than delayed intervention after NMJ deterioration has progressed.

#### Adjust interpretation accordingly

Apply predicted decoupling risk to grip strength interpretation. Do not assume grip improvement equals mortality risk reduction, especially for narrow-target therapies.

#### Monitor mass-to-strength ratio

In patients receiving anabolic therapy, track both mass and strength independently. Disproportionate mass gains without strength improvement suggests NMJ-limited response (therapeutic paradox).

#### Use complementary biomarkers

Never rely on grip strength alone in gene therapy patients. Table 3 provides illustrative, therapy-specific suggestions for complementary assessments rather than prescriptive panels.

**TABLE 3:** Therapy-specific interpretation guide and recommended complementary markers.

Therapy	Grip interpretation	Complementary markers	Rationale
Follistatin	Target engagement only; not mortality proxy	Gait speed, VO_2_max, DXA, CRP, cognition	Triangulates systems unaffected by follistatin (McPherron et al., 1997; Latres et al., 2015)
Klotho	Reasonable mortality proxy; may underestimate benefit	Cognition, PWV, phosphate/calcium	Confirms multi-system engagement (Kuro-o et al., 1997; Kuro-o, 2019)
FOXO3	Reasonable mortality proxy	Oxidative stress markers, HbA1c, autophagy	Assesses cellular stress resistance (Willcox et al., 2008; Martins et al., 2016)
hTERT	Long-term trajectory more informative	Telomere length, senescence markers	Confirms target engagement (Bernardes de Jesus et al., 2012; Mojiri et al., 2021)
SIRT1	Reasonable mortality proxy	HbA1c, lipids, NAD^+^ levels	Assesses metabolic regulation (Imai and Guarente, 2014; Satoh et al., 2013)
PGC-1α	Reflects metabolic capacity; other systems unassessed	VO_2_max, lactate threshold, mitochondrial markers	Confirms mitochondrial enhancement (Handschin and Spiegelman, 2008; Lin et al., 2002)
VEGF	Context-dependent; use only in ischemic patients	ABI, perfusion imaging, exercise tolerance	Assesses vascular improvement (Celletti et al., 2001)
FGF21	Reasonable mortality proxy; pair with metabolic markers	Glucose, lipids, body composition, liver function	Confirms metabolic improvement (Geng et al., 2020; Kharitonenkov and DiMarchi, 2015)

PWV, pulse wave velocity; ABI, ankle-brachial index; DXA, dual-energy X-ray absorptiometry. Complementary markers should be selected based on therapy mechanism and patient-specific considerations. Superscript numbers indicate supporting references.

## Discussion

Grip strength has earned its reputation as a powerful biomarker of biological aging through decades of epidemiological research. That reputation, however, was established in a world where interventions capable of improving grip strength were inevitably broad in their effects—exercise, nutrition, and disease management all influence multiple aging-sensitive systems simultaneously.

The gene therapy era disrupts this assumption. For the first time, we possess interventions capable of substantially improving grip strength through targeted molecular mechanisms that leave other aging processes unaddressed. This creates both opportunities and pitfalls. Appropriately interpreted, grip strength remains a valuable clinical tool—but its interpretation must account for the specific interventions a patient has received.

The neuromuscular junction emerges as a critical and often-overlooked determinant of functional strength. The observation that strength loss outpaces mass loss by two-to fivefold reflects NMJ deterioration that sets a ceiling on the functional benefits achievable through muscle-targeted interventions alone. Follistatin’s efficacy as a functional enhancer—not merely a mass enhancer—depends critically on preserved NMJ integrity, creating a therapeutic paradox in patients with advanced neural deterioration.

The clearest legitimate application of anabolic gene therapy is not longevity optimization but cancer-associated cachexia—a syndrome affecting the majority of patients with advanced cancer, contributing directly to a substantial fraction of cancer deaths, and for which no anabolic therapy is currently approved (Baracos et al., 2018). In that setting the therapeutic objective is preservation of muscle mass, strength, and function themselves, and grip is read as a direct treatment effect rather than a mortality surrogate; the biomarker-decoupling caution developed here is therefore largely orthogonal to the cachexia indication and becomes salient only when the same intervention is applied to asymptomatic individuals for longevity. Recognizing this distinction sharpens rather than limits the framework.

Single-system therapies such as follistatin carry high decoupling risk; multi-system therapies such as klotho, FOXO3, and SIRT1 may produce grip changes that more accurately reflect systemic benefit while potentially preserving the neural infrastructure required for functional translation. Context matters: VEGF gene therapy is appropriate for documented ischemic disease but carries an unfavorable risk-benefit profile for healthy longevity applications (Celletti et al., 2001; Carmeliet, 2000). hTERT, within the bounds of current *in vitro* and animal evidence, has not shown oncogenic transformation when delivered exogenously in normal cells and may, in principle, lower cancer-promoting signals through senescence reduction, although long-term human safety data remain to be established (Bodnar et al., 1998; Morales et al., 1999; Jiang et al., 1999). Timing matters: pre-emptive intervention before significant NMJ deterioration (ages 50–55) may achieve substantially better outcomes than delayed intervention.

The broader lesson extends beyond grip strength to the fundamental challenge of measuring biological age in an era of targeted interventions. As we develop increasingly precise tools to modify specific aging pathways, we must develop correspondingly sophisticated frameworks for interpreting the biomarkers those interventions affect. The goal of longevity medicine is not to optimize biomarkers but to extend healthy human lifespan—a goal that requires maintaining clear sight of what our measurements actually mean.

## Data Availability

The original contributions presented in this study are included in the article. No new datasets were generated or analyzed; all data discussed derive from the cited published literature. Further inquiries can be directed to the corresponding author.
